# Qualitative and Quantitative Detection of Typical Reproductive Hormones in Dairy Cows Based on Terahertz Spectroscopy and Metamaterial Technology

**DOI:** 10.3390/molecules29102366

**Published:** 2024-05-17

**Authors:** Shuang Liang, Jingbo Zhao, Wenwen Zhao, Nan Jia, Zhiyong Zhang, Bin Li

**Affiliations:** 1 Intelligent Equipment Research Center, Beijing Academy of Agriculture and Forestry Sciences, Beijing 100097, China; 13803484013@163.com (S.L.); 13707665265@163.com (J.Z.); zhaoww@nercita.org.cn (W.Z.); jia_nan0120@sina.com (N.J.); 2 College of Agricultural Engineering, Shanxi Agricultural University, Taigu 030801, China; zzyzzy1979@163.com

**Keywords:** Terahertz, metamaterial, qualitative and quantitative

## Abstract

Progesterone (PROG) and estrone (E_1_) are typical reproductive hormones in dairy cows. Assessing the levels of these hormones in vivo can aid in estrus identification. In the present work, the feasibility of the qualitative and quantitative detection of PROG and E_1_ using terahertz time-domain spectroscopy (THz-TDS) and metamaterial technology was preliminarily investigated. First, the time domain spectra, frequency domain spectra, and absorption coefficients of PROG and E_1_ samples were collected and analyzed. A vibration analysis was conducted using density functional theory (DFT). Subsequently, a double-ring (DR) metamaterial structure was designed and simulated using the frequency domain solution algorithm in CST Studio Suite (CST) software. This aimed to ensure that the double resonance peaks of DR were similar to the absorption peaks of PROG and E_1_. Finally, the response of DR to different concentrations of PROG/E_1_ was analyzed and quantitatively modeled. The results show that a qualitative analysis can be conducted by comparing the corresponding DR resonance peak changes in PROG and E_1_ samples at various concentrations. The best R^2^ for the PROG quantitative model was 0.9872, while for E_1_, it was 0.9828. This indicates that terahertz spectral–metamaterial technology for the qualitative and quantitative detection of the typical reproductive hormones PROG and E_1_ in dairy cows is feasible and worthy of in-depth exploration. This study provides a reference for the identification of dairy cow estrus.

## 1. Introduction

**Figure 1:**
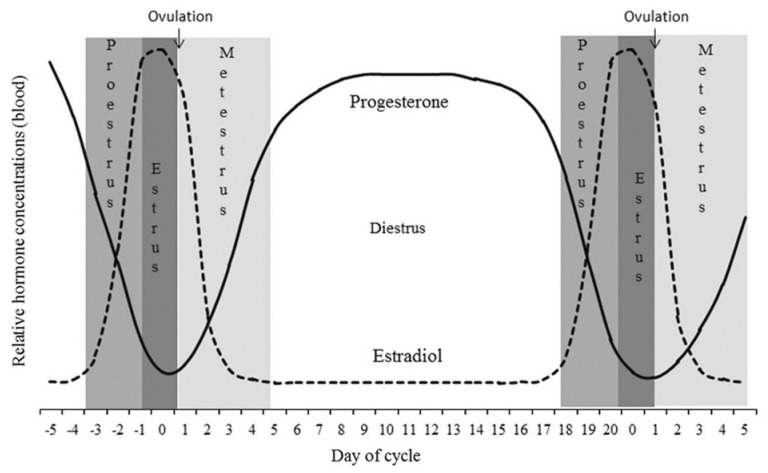
Changes in reproductive hormone content during the estrus cycle in cows.

Estrus identification is crucial for breeding management in dairy farms as it directly affects the calving interval, reproductive ability, and milk production. Timely and accurate estrus identification holds significant importance for dairy farming [1]. Currently, estrus identification relies on manual detection, which includes a vaginal examination and tail wax coating. However, these methods are typically labor-intensive, subjective, and inefficient [2]. During the estrus stage, cows generally exhibit changes in their physiological and behavioral characteristics, such as temperature fluctuations [3,4], increased movement [5,6], and mounting behavior [7]. Researchers have developed a variety of wearable devices based on these characteristics and have implemented them in small-scale applications. However, these devices still have shortcomings, such as their susceptibility to damage and detachment, alongside the distress caused to the animals wearing them. Previous studies have shown that the physiological process of estrus in dairy cows is regulated by hormones in the hypothalamus, anterior pituitary, ovaries, and uterus (Figure 1) [8]. Therefore, methods for estrus identification based on changes in typical reproductive hormone levels in the body are worth exploring.

Hormones are bioactive substances secreted directly into the bloodstream by the endocrine organs or tissues of humans and animals. The coordinated actions of multiple hormones are necessary to maintain the body’s metabolism and functions. Reproductive hormones play an important role in the development, function, and regulation of the reproductive system. Researchers have conducted quantitative studies on reproductive hormones based on ELISA [9], gas chromatography [10], and high-performance liquid chromatography [11]. Although these methodologies are efficacious, they come with significant constraints, including time-intensive and costly procedures, and the requirements of skilled personnel and strict adherence to experimental conditions. Therefore, there is a need for the development of a rapid and accurate method for detecting typical reproductive hormones.

Spectral analysis technology has been widely used in hormone detection and research because of its low detection cost, nondestructive properties, and speed [12,13,14,15]. As compared with other spectra, the terahertz (THz) spectrum is non-ionizing, has high penetrability, and can provide intermolecular information on the vibrational activities of molecules. It can detect weak intermolecular interactions such as hydrogen bonds and van der Waals forces. Since the vibration frequency of hormone molecules falls within the terahertz band, terahertz spectroscopy can be utilized for their qualitative and quantitative analysis. Combined with terahertz spectroscopy, metamaterial technology, a signal enhancement technology, has recently emerged as a method for qualitative and quantitative detection [16,17,18,19]. Uro et al. [20] studied the terahertz absorption characteristics of melatonin in the 1.5 THz to 4.5 THz range, identifying strong absorption peaks at 3.21 THz and weak absorption peaks at 4.20 THz. They explained the formation of absorption peaks based on DFT. Shen [21] studied the vibration characteristics of melatonin, obtained its spectrum at 0.518 THz, and calculated and analyzed its vibration characteristics. Shen found that low-frequency vibration mainly arose from intermolecular and intramolecular vibration coupling, while high-frequency vibration originated from intramolecular vibration. Zhu [22] studied the absorption characteristics of dopamine in the terahertz band, discovering multiple absorption peaks in the range of 0.5 to 18 THz, and identified and analyzed its vibration modes based on DFT. Yeon [23] studied the terahertz spectral characteristics of stable and metastable progesterone polycrystalline structures and was able to distinguish between different states of progesterone. However, the aforementioned studies solely utilized terahertz spectroscopy for qualitative research on these hormones, indicating a gap in quantitative analysis modeling research combined with hormone terahertz fingerprint spectra.

Inefficient estrus detection causes low pregnancy rates, resulting in economic losses and the deterioration of fertility performance [24]. The physiological characteristics of cow estrus, namely the typical bovine reproductive hormones PROG and E_1_, were chosen as the research objects in this study, with the aim of reducing these issues. Terahertz spectral–metamaterial technology was explored as a method for the qualitative and quantitative detection of PROG and E_1_, aiming to provide a new reference method for cow estrus identification.

## 2. Materials and Methods

### 2.1. Sample Preparation

In this study, progesterone (PROG, purity > 98%, CAS 57-83-0) and estrone (E_1_, purity > 98%, CAS 56-17-3) were purchased from Shanghai Yuanye Biotechnology Co., LTD, Shanghai, China, and anhydrous ethanol (Analytical Reagent, 99.7%) was purchased from Shanghai Yien Chemical Technology Co., LTD, Shanghai, China. Neither the reagents nor the solutions underwent additional purification prior to usage.

Three pressed tablets were prepared for both PROG and E_1_ for terahertz absorption characterization. The tablet sample preparation process was as follows: Initially, 100 mg each of PROG/E_1_ and polyethylene powders were weighed using an electronic balance and then thoroughly mixed after grinding. Next, the powder was poured into a tablet grinding tool, with attention paid to preventing powder spillage, and pressure was applied to the tablets for 5 min at 5 t to ensure a smooth surface. Finally, the tablets were removed, and their thickness was measured using digital calipers.

The test solution was prepared as follows: Initially, 80 mg of PROG/E_1_ powder was weighed using an electronic balance. An amount of 2 mL of ethanol solution was measured with a measuring cylinder. The sample powder and ethanol solution were transferred to a beaker and stirred thoroughly to prepare the test solution at a concentration of 40 mg/mL. Next, 1 mL of the 40 mg/mL solution was transferred into a test tube, and 1 mL of ethanol was added to adjust the concentration to 20 mg/mL. Finally, the solution was prepared with a gradient of concentrations, namely 5, 10, 20, and 40 mg/mL.

### 2.2. Metamaterial Structure Design

**Figure 2:**
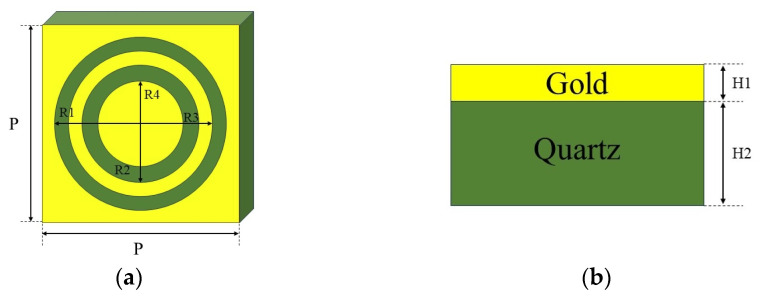
Metamaterial structure diagram: (**a**) double-ring structure and (**b**) thickness.

Metamaterials are artificial materials distinguished by their distinctive electromagnetic properties, stemming from a structure comprising regularly spaced subwavelength elements [25,26]. Notably, metamaterials exhibit significant field enhancement effects, thereby facilitating the detection of extremely low concentrations of both chemical and biological substances. Here, the design and simulation of metamaterial structures were carried out based on the frequency domain solution algorithm in CST. The metamaterial structure was a double-ring structure, consisting of a double-ring metal array periodically arranged on a quartz substrate, as shown in Figure 2a. The metal was gold, with a thickness of 0.2 μm. The thickness of the quartz was set to 10 μm to avoid the effect of echoes in the simulation. The actual thickness was 500 μm, and the period was P = 100 μm. The metamaterial structure was designed using CST Studio Suite software and simulated in the frequency range of 0 to 1.5 THz.

### 2.3. THz-TDS Measurements

**Figure 3:**
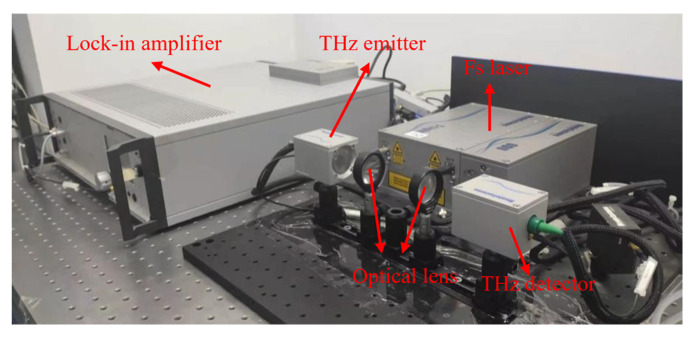
Diagram of the THz-TDS system.

The THz-TDS system used in this study, produced by Menlo, Martinsried, Germany (Figure 3), pumps a laser with a wavelength of 1560 nm, a repetition frequency of 100 MHz, a measurement range of 0.1–2.5 THz, and a spectral resolution of less than 1.2 GHz [27,28].

The system is located in an airtight enclosure. In order to avoid the influence of water vapor during the measurement process, the ambient temperature is maintained at around 25 °C; dry air is continuously circulated to keep the ambient humidity below 7%. As the terahertz pulse is repeatedly reflected at the sample–air interface, an echo is generated in the terahertz time-domain spectrum. By selecting the window before the echo arrives as the effective time-domain waveform and setting all the terahertz pulses after this window to 0, and then using the Fourier transform to convert the time-domain signal into a frequency-domain signal, the calculation formula is as follows:

(1) $$\tilde{E}(\omega )=A(\omega )e^{-i\phi (\omega )}=\int dtE(t)e^{-i\omega t}$$

The absorption coefficients of the samples to be tested are as follows:

(2) $$n(\omega )=\frac{c\varphi (\omega )}{d\omega }+1$$

(3) $$\alpha (\omega )=\frac{d}{2}ln\frac{4n(\omega )}{A(\omega ){\left(n\left(\omega \right)+1\right)}^{2}}+1$$

The transmission coefficient is obtained by comparing the sample spectra with the reference signal spectra using the following formula:

(4) $$T(\omega )=A_{s}/A_{r}$$

where ϕ(ω) denotes the phase of the electric field, A(ω) denotes the amplitude of the electric field, E(t) denotes the electric field, d denotes the thickness of the sample, c denotes the speed of light in a vacuum, ω denotes angular frequency, $A_{s}$ denotes the amplitude of the sample frequency-domain signal, and $A_{r}\;$denotes the amplitude of the reference frequency-domain signal.

The spectra of the tablet samples were collected by placing the pressed samples in the sample holder, placing them in the terahertz optical path to collect their time-domain spectral signals, and then calculating their absorption coefficients according to Equations (1)–(3). The spectra of the test solution were collected by extracting 5 μL of the solution from the surface of the metamaterials using a pipette gun and then drying it in a dryer until the ethanol solution evaporated completely. At this time, the surface of the metamaterials was covered with a layer of PROG/E_1_ film. The time-domain spectral signals were collected, and the transmittance spectra were calculated according to Equations (1) and (4).

**Figure 4:**
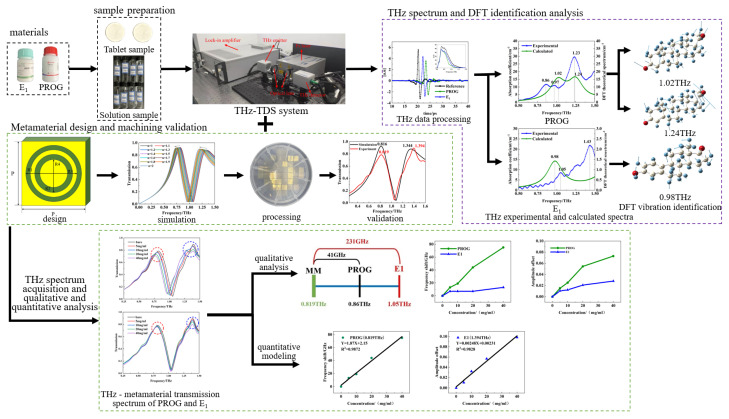
Flow chart of research.

The experimental flow chart is shown in Figure 4. Initially, the terahertz spectra of PROG and E_1_ were collected and analyzed; they were then compared with the results of DFT, and their vibration modes were identified. Next, the metamaterial structure was designed by combining the terahertz fingerprint spectra of PROG and E_1_. Subsequently, numerical simulations and real-world machining tests were conducted. Finally, a concentration gradient PROG–ethanol solution and E_1_–ethanol solution was prepared. Then, the terahertz metamaterial was employed for detection, and the responses of PROG and E_1_ to the resonant peak were qualitatively and quantitatively analyzed.

## 3. Results

### 3.1. THz Spectroscopy and DFT Analysis of Samples

#### 3.1.1. Terahertz Spectral Measurements of the Samples

**Figure 5:**
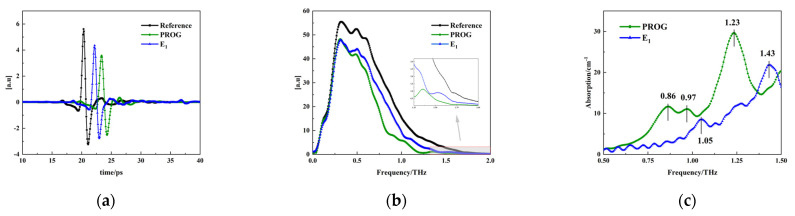
Terahertz spectral information: (**a**) time domain, (**b**) frequency domain, and (**c**) absorption coefficient.

The THz spectrum of air was used as the reference signal, and the THz spectra of PROG and E_1_ were used as the sample signals. Due to the effect of instrumental noise, the frequency bands with the highest signal-to-noise ratios were selected for analysis. Figure 5a,b show the terahertz time-domain spectra and frequency-domain spectra of dry air and of the samples, respectively. It can be seen that the sample signal shows a time delay of about 2~3 ps compared with the reference signal. This is due to the different propagation speeds of terahertz waves in the air and in the samples. The amplitude of the sample signal shows different degrees of attenuation compared to that of the reference signal, which is due to the absorption, reflection, and scattering of the samples.

After converting the time-domain signals to frequency-domain signals and smoothing them, the sample signals all showed an attenuation in intensity compared with the reference signals. The frequency-domain signals of PROG were weaker than those of estrone, indicating that the absorption of terahertz waves by PROG was stronger than that by E_1_. The absorption characteristics of PROG and E_1_ are depicted in Figure 5c. PROG displays weak absorption peaks at 0.86 THz and 0.97 THz, accompanied by a prominent absorption peak at 1.23 THz. Conversely, E_1_ exhibits a weak absorption peak at 1.05 THz and a strong absorption peak at 1.43 THz.

#### 3.1.2. Comparative Analysis of DFT Calculation and Experimental Results of PROG and E_1_

**Figure 6:**
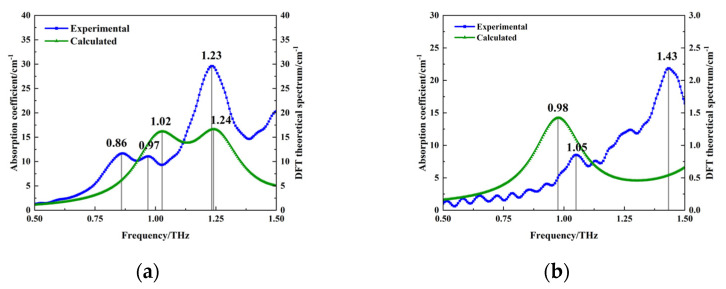
Comparison of experimental and calculated results: (**a**) PROG and (**b**) E_1._

To obtain the molecular vibrational modes corresponding to the absorption peaks of PROG and E_1_, geometrical configuration optimization and theoretical vibrational frequency calculations of PROG and E_1_ molecules were carried out using density functional theory (DFT) regarding the B3LYP hybridization flooding, combined with the 6-311G basis group, ensuring that no imaginary frequencies appeared during the calculations. Figure 6a shows the calculated and experimental results for PROG; calculated absorption peaks can be seen at 1.02 THz and 1.24 THz, and experimental peaks appear at 0.86 THz, 0.97 THz, and 1.23 THz. Figure 6b shows the calculated and experimental results for E_1_; the calculated absorption peak can be seen at 0.98 THz, and experimental peaks are located at 1.05 THz and 1.43 THz.

Comparing the theoretical calculations and experimental results, it is evident that some absorption peaks, such as the absorption peaks at 0.86 THz for PROG and 1.43 THz for E_1_, are not reflected in the theoretical calculations. This discrepancy may be related to the isolated single-molecule model used in the calculations, which only considered intramolecular vibration modes and did not take into account intermolecular interactions, the crystal field effect, and crystal resonance [29]. Furthermore, there is a deviation between the theoretical calculations and experimental results. An absorption peak at 1.24 THz was obtained using PROG calculations, while the experiments yielded 1.23 THz. The difference in temperature may have caused this phenomenon, as the measurements were taken at ambient temperature, while the theoretical calculations were based on a temperature of 0 K. Thermal expansion and contraction may have led to a change in bond lengths, resulting in a frequency shift [30].

#### 3.1.3. Vibration Mode Analysis and Absorption Peak Identification

**Figure 7:**
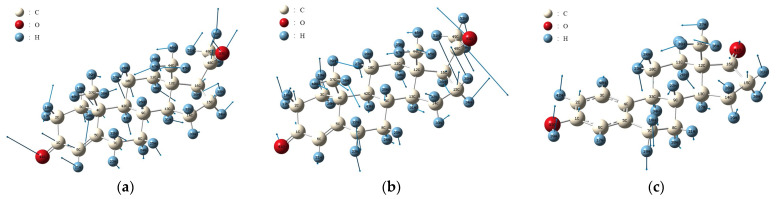
Vibration identification of (**a**) PROG at 1.02 THz and (**b**) 1.24 THz; (**c**) E_1_ at 0.98 THz.

Figure 7 depicts the molecular vibrational modes of the PROG absorption peaks at 1.02 THz and 1.24 THz, as well as of the E_1_ absorption peak at 0.98 THz. The PROG absorption peak at 1.02 THz is mainly due to the wobbling vibrations of the 5C-6C-1C-2C-3C and 12C-13C-14C-15C-16C atoms, hydrogen atoms, oxygen atoms, and the 48CH3 methyl group. The absorption peak at 1.24 THz originates from the overall torsional vibration of the 5C-6C-1C-2C-3C and 12C-13C-14C-15C-16C atom groups, the torsional vibration of the external hydrogen atoms, the carboxylate group, and the overall torsional vibration of the methyl group. The absorption peak at 0.98 THz for E_1_ mainly originates from the overall rocking vibrations of the atomic groups 6C-1C-2C-3C and 14C-15C-16C and the outer band of hydrogen atoms, as well as the overall rocking vibration of the 35CH3 methyl group.

### 3.2. Simulation and Verification of Metamaterials

**Figure 8:**
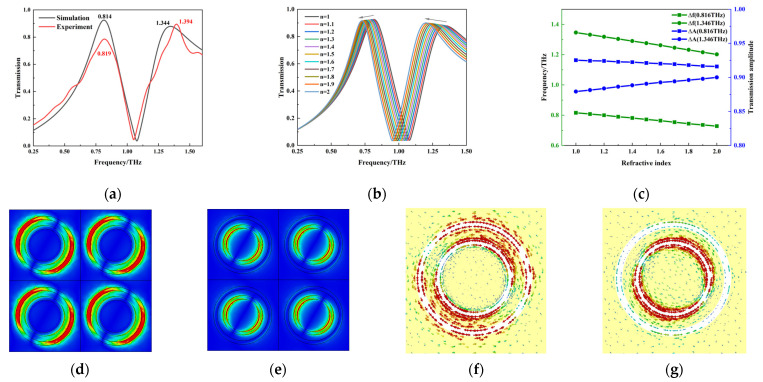
Metamaterial design and simulation; (**a**) simulation and test results; (**b**) responses to different refractive indices; (**c**) relationship between resonant peak frequency shifts, intensity changes, and the refractive index; (**d**) electric field distribution at 0.819 THz; (**e**) electric field distribution at 1.394 THz; (**f**) surface current distribution at 0.819 THz; (**g**) surface current distribution at 1.394 THz.

The metamaterial simulation was carried out using the frequency domain solver in CST software. The terahertz wave was vertically incident on the surface of the structure along the *Z*-axis direction in the simulation, with the X and Y directions set as the periodic boundary conditions and the Z direction set as the open boundary condition. Figure 8a shows a comparison between the DR transmission curve simulation and the actual test results. It can be seen that the two trends are consistent, but there are small differences between the position and intensity of the resonance peaks, which may be related to the processing accuracy. In addition, there are two DR resonance peaks with frequencies of 0.819 THz and 1.394 THz, which are similar to the absorption peaks of 0.86 THz and 1.43 THz for PROG and E_1_, respectively.

In order to investigate the DR’s ability to detect substances, its response to substances with different refractive indices was simulated, and the results are shown in Figure 8b,c. The two resonance peaks show a good linear relationship between frequency and intensity with concentration changes. In addition, in order to illustrate the physical mechanism of DR resonance, the electric field and current distribution of DR at the resonance peaks are shown in Figure 8d–g. It can be seen that the resonance mode at the DR resonance peaks is dipole resonance. Therefore, the feasibility of PROG and E_1_ detection using DR was demonstrated by this simulation.

### 3.3. Measurement and Analysis of PROG and E_1_ THz Spectra

#### 3.3.1. Measurement of PROG and E_1_ THz Spectra

**Figure 9:**
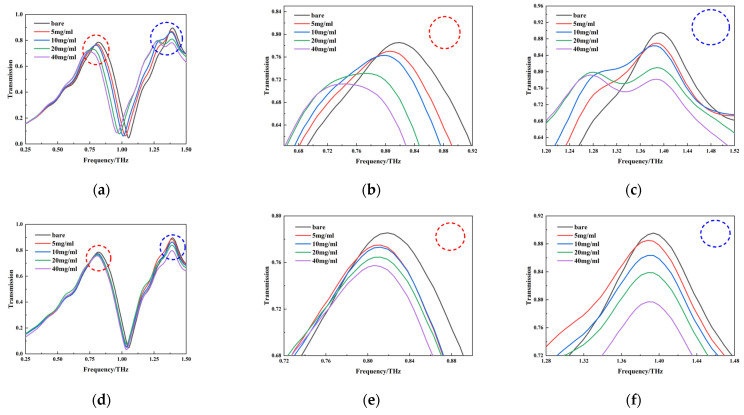
Transmission spectra of different concentrations of PROG and E_1_; (**a**) PROG transmission spectra; (**b**,**c**) detailed spectrum of transmission peak at 0.819 THz and 1.394 THz for PROG; (**d**) E_1_ transmission spectra; (**e**,**f**) detailed spectrum of transmission peak at 0.819 THz and 1.394 THz for E_1._

Samples of the test solutions were examined using the terahertz–metamaterials technique. The transmission spectra of blank metamaterials and of the four concentrations of PROG and E_1_ solutions (5 mg/mL, 10 mg/mL, 20 mg/mL, and 40 mg/mL) are represented in Figure 9a–c and Figure 9d–f, respectively; each curve represents the average of three measurements.

Regarding PROG detection, it can be seen from Figure 9a that the resonance peaks at 0.819 THz and 1.394 THz both underwent significant changes. The resonance peak at 0.819 THz shows a significant redshift phenomenon with an increase in PROG concentration; there is a redshift from 0.819 THz to 0.744 THz, with a decrease of 0.0792 in transmittance intensity (Figure 9b). The resonance peak at 1.394 THz increases with PROG concentration and has no frequency shift, and transmission intensity decreases by 0.1136 (Figure 9c). As for E_1_ detection, as shown in Figure 9d, the two resonance peaks show almost no frequency shift, but the transmission intensity changes; the transmission intensity of the resonance peaks at 0.819 THz and 1.394 THz decreases by 0.028 (Figure 9e) and 0.0983 (Figure 9f), respectively.

By analyzing the PROG and E_1_ transmittance spectra of the metamaterials, it was found that changes in their concentrations are reflected in the frequency shifts and transmitted intensity changes in the resonance peaks, which provide a theoretical basis for qualitative and quantitative analysis. There are two main reasons for the resonance peak frequency shift and intensity change. Firstly, with an increase in sample concentration, the film on the surface of the metamaterial thickens, and the absorption of the terahertz wave becomes stronger. This leads to a reduction in the transmitted terahertz wave, which is manifested as a reduction in resonance peak intensity. In metamaterials, resonance peak frequency shifts are usually caused by changes in the effective refractive index of the surrounding materials. When PROG and E_1_ cover the surface of the metamaterial, the effective refractive index changes, leading to a frequency shift in the resonance peak. In addition, the frequency shift of the resonance peak at 1.394 THz is not obvious in this study, which may be due to the sample concentration not being high enough to change the effective refractive index and thus failing to cause a resonance peak frequency shift in the metamaterial [31].

#### 3.3.2. Qualitative Analysis of PROG and E_1_

**Figure 10:**
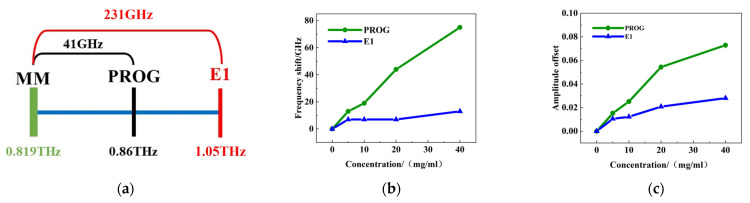
Qualitative analysis: (**a**) matching the degree of the PROG/E_1_ absorption peak with the metamaterial resonance peak, (**b**) frequency change om the 0.819 THz resonance peak, and (**c**) intensity change.

The analysis above suggests that the alterations in the resonance peak at 0.819 THz are particularly pronounced in metamaterials. This result was therefore utilized for qualitative assessment. The discrepancy between the absorption peak of PROG at 0.86 THz and the resonance peak at 0.819 THz is 41 GHz, while the difference between the absorption peak of E_1_ at 1.05 THz and the resonance peak is 231 GHz (Figure 10a). This indicates that the absorption peak frequency of PROG is closer to the resonance peak frequency of the metamaterial. Figure 10b,c show that PROG exhibits a greater frequency shift and intensity variation compared to E_1_. Consequently, focusing solely on the variation in the resonance peak at 0.819 THz is sufficient. The substance that exhibits a larger frequency shift and intensity variation also has the closest absorption peak to the resonance peak of the metamaterials, thus facilitating the preliminary qualitative analysis of PROG and E_1_.

#### 3.3.3. Quantitative Analysis of PROG and E_1_

The response of the metamaterial resonance peaks to the different concentrations of PROG is summarized in Table 1. It is evident that both the frequency and intensity of the resonance peaks fluctuate with the concentration of PROG; therefore, a quantitative modeling study was undertaken.

**Figure 11:**
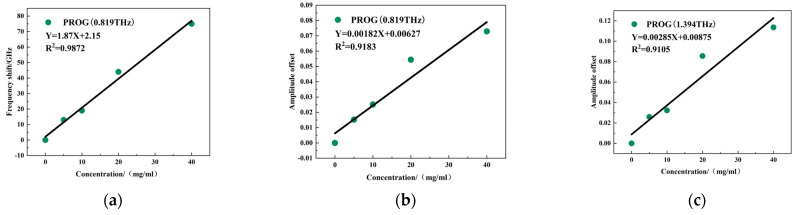
Resonance peak change: (**a**) 0.819 THz frequency shift, (**b**) 0.819 THz intensity change, and (**c**) 1.394 THz intensity change and PROG concentration linear fitting results.

Figure 11a,b show the results of quantitative modeling between the frequency shift and intensity change of the metamaterial resonant peak at 0.819 THz and PROG concentration. The linear regression equation for the frequency shift is Y = 1.87X + 2.15, with R^2^ = 0.9872, and the linear regression equation for the intensity change is Y = 0.00182X + 0.00627, with R^2^ = 0.9183. Figure 11c shows the relationship between the intensity of the resonance peak at 1.394 THz and the concentration of PROG, with a linear regression equation of Y = 0.00285X + 0.00875 and R^2^ = 0.9105. It can thus be seen that the quantitative detection of PROG was achieved using the variation in the resonance peak at 0.819 THz and the 1.394 THz model, and each R^2^ was above 0.90.

Table 2 summarizes the response of the metamaterial resonance peaks to the different concentrations of E_1_, which primarily manifested as a decrease in the intensity of the resonance peaks as E_1_ concentration increases, thus suggesting the feasibility of conducting a quantitative modeling study.

**Figure 12:**
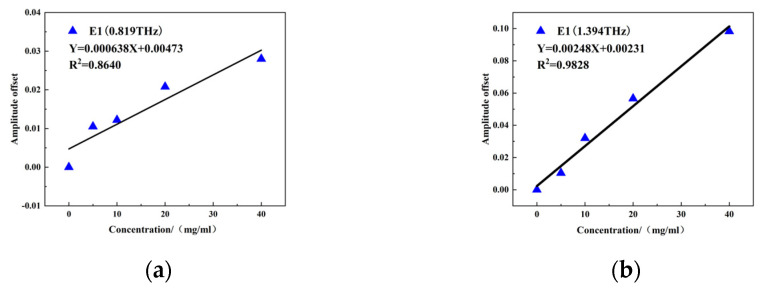
Resonance peak change: (**a**) 0.819 THz intensity change and (**b**) 1.394 THz intensity change and E_1_ concentration linear fitting results.

Figure 12a,b show the quantitative modeling results for the variations in resonant peak intensity at 0.819 THz and 1.394 THz, respectively. Both resonance peaks show a good linear relationship with E_1_ concentration. The linear regression equation of the intensity change at 0.819 THz is Y = 0.000638X + 0.00473, with R^2^ = 0.8640. The linear regression equation of the intensity change at 1.394 THz is Y = 0.00248X + 0.00231, with R^2^ = 0.9828. The superior fitting degree between the intensity and concentration change at 1.394 THz is probably due to the smaller frequency offset (36 GHz) between the absorption peak at 1.43 THz and the resonant peak at 1.394 THz of E_1_, which is smaller than the frequency offset (161 GHz) between the absorption peak at 0.98 THz and the resonant peak at 0.819 THz of E_1_. The optimal E_1_ quantitative detection model was therefore constructed using the changes in resonant peaks at 0.819 THz and 1.394 THz, and R^2^ was above 0.85.

In summary, through comparing the response of the resonant peak at DR 0.819 THz to different concentrations of PROG and E_1_, it was observed that the response of DR to PROG is more pronounced. This may be attributed to the fact that the absorption peak of PROG at 0.86 THz is closer to the resonant peak frequency of DR. Subsequently, a quantitative PROG and E_1_ detection model was established. It was revealed that a linear relationship exists between the frequency shift or intensity change of DR and the concentration of PROG and E_1_, with an R2 value above 0.85. Additionally, an intriguing observation was made: the quantitative PROG detection model based on the resonance peak at 0.819 THz outperforms that based on 1.394 THz, while the quantitative E_1_ detection model based on the resonance peak at 1.394 THz surpasses that based on 0.819 THz. This could be attributed to the frequency offset between the absorption peaks of PROG and E_1_ and the resonant peak of DR. Employing metamaterial sensors with similar absorption peaks to the target substances may yield a more discernible response and enhance detection efficiency.

In conclusion, the qualitative and quantitative detection of hormones can preliminarily be achieved through the utilization of terahertz spectrum–metamaterial technology. In contrast to previous studies employing metamaterials [32,33], our approach involves designing the metamaterial structure according to the hormone absorption peaks, thereby achieving resonance coupling. The results demonstrate that this method can not only facilitate quantitative detection but can also enable a preliminary qualitative analysis.

## 4. Conclusions

In this paper, a double-ring metamaterial structure was designed for the qualitative and quantitative detection of the typical dairy cow reproductive hormones, PROG and E_1_. Firstly, the terahertz characteristic spectra of PROG and E_1_ were analyzed, and a vibration mode identification analysis was performed based on the Gaussian density functional (DFT-B3LYP) theory. Then, a metamaterial structure with double-resonant peaks was designed based on the absorption characteristics of PROG and E_1_, and the response of the structure to different refractive indices was confirmed. Finally, a concentration gradient of PROG/E_1_–ethanol solution was prepared, and the changes in the metamaterial double-resonance peaks were utilized for qualitative and quantitative analysis. The offset of the metamaterial resonance peaks and PROG and E_1_ absorption peaks was calculated, and a qualitative analysis was conducted by observing the frequency shifts and intensity changes in PROG and E_1_ samples at various concentrations. Based on the frequency shifts and intensity changes in the resonant peaks, quantitative models for PROG and E_1_ detection were constructed to facilitate quantitative analysis. The results show that a preliminary qualitative and quantitative detection of hormones can be achieved using terahertz spectrum–metamaterial technology, providing a new method for cow estrus identification.

## Figures and Tables

**Table 1:** Effects of different concentrations of PROG on metamaterial resonance peaks.

PROG	0.819 THz		1.394 THz
	Frequency Shift/GHz	Amplitude Offset	Amplitude Offset
5 mg/mL	13	0.0152	0.0259
10 mg/mL	19	0.0251	0.0323
20 mg/mL	44	0.0544	0.0855
40 mg/mL	75	0.0729	0.1136

**Table 2:** Effects of different concentrations of E_1_ on metamaterial resonance peaks.

E_1_	0.819 THz	1.394 THz
	Amplitude Offset	Amplitude Offset
5 mg/mL	0.0105	0.0104
10 mg/mL	0.0122	0.0320
20 mg/mL	0.0208	0.0566
40 mg/mL	0.0280	0.0983

## Data Availability

The original contributions presented in the study are included in the article, further inquiries can be directed to the corresponding author.
